# Supplementary material for: Empirical methods for controlling false positives and estimating confidence in ChIP-Seq peaks
Source: BMC Bioinformatics. 2008 Dec 5;9:523. doi: 10.1186/1471-2105-9-523 (PMC2628906; doi:10.1186/1471-2105-9-523)
Supplement: Additional File 1 — A variety of html documents from the USeq web site detailing the available applications, their best usage, output file type descriptions, command line menus, etc. [file 1471-2105-9-523-S1.zip › USeqUserGuides/JavaDoc.zip!/JavaDoc/util/bio/parsers/UCSCGeneModelTableReader.html]

UCSCGeneModelTableReader


|  |  |  |
| --- | --- | --- |
| **Overview** \| **Package** \| **Class** \| **Use** \| **Tree** \| **Deprecated** \| **Index** \| **Help** | |  |
| **PREV CLASS**   NEXT CLASS | **FRAMES**    **NO FRAMES**     **All Classes** |  |
| SUMMARY: NESTED \| FIELD \| CONSTR \| METHOD | DETAIL: FIELD \| CONSTR \| METHOD |  |


---


## util.bio.parsers Class UCSCGeneModelTableReader

```
java.lang.Object
  util.bio.parsers.UCSCGeneModelTableReader
```

---

``` public class UCSCGeneModelTableReader extends java.lang.Object ```

Parses a UCSC gene model table file (either refSeq or refFlat), use table options for #hg17.refGene.name hg17.refLink.product hg17.geneName.name hg17.refSeqSummary.summary... example:
#refGene.name refGene.chrom refGene.strand refGene.txStart refGene.txEnd refGene.cdsStart refGene.cdsEnd refGene.exonCount refGene.exonStarts refGene.exonEnds refLink.product geneName.name refSeqSummary.summary
NM\_198576 chr1 + 995569 1031415 995619 1030284 36 995569,997647,1010723,1016111,1016522,1016827,1017258,1018541,1018840,1019125,1019411,1019636,1020463,1020661,1021035,1021266,1021462,1021699,1022122,1022629,1022875,1023078,1023314,1024169,1024538,1024868,1025205,1025535,1025729,1026028,1026555,1026755,1027030,1029055,1029750,1030126, 995820,997909,1010771,1016327,1016747,1017052,1017465,1018760,1019035,1019326,1019560,1019742,1020580,1020826,1021179,1021391,1021568,1022038,1022260,1022757,1022990,1023198,1023668,1024362,1024754,1025098,1025340,1025632,1025894,1026140,1026672,1026948,1027118,1029280,1029854,1031415, agrin AGRIN Agrin is a neuronal aggregating factor that induces the aggregation of ... \*

---

**Constructor Summary**

`UCSCGeneModelTableReader()`

`UCSCGeneModelTableReader(java.io.File file,
int numToSubtractFromEnd)`


| **Method Summary** | |
| --- | --- |
| `java.util.HashMap<java.lang.String,UCSCGeneLine[]>` | `getChromSpecificGeneLines()` |
| `UCSCGeneLine[]` | `getGeneLines()` |
| `static void` | `main(java.lang.String[] args)` |
| `void` | `parseGeneTableFile(java.io.File file, int numToSubtractFromEnd)` |
| `void` | `printAllExons(int bpEndMod)`             Prints all exons to standard out. |
| `void` | `readOutHash()`             To test your hash. |
| `void` | `setGeneLines(UCSCGeneLine[] geneLines)` |
| `void` | `splitByChromosome()`             Splits a UCSCGeneLine[] by chromosome into a HashMap of chromosome:UCSCGeneLine[]. |

**Methods inherited from class java.lang.Object**

`equals, getClass, hashCode, notify, notifyAll, toString, wait, wait, wait`

**Constructor Detail**

### UCSCGeneModelTableReader

```
public UCSCGeneModelTableReader(java.io.File file,
                                int numToSubtractFromEnd)
```

**Parameters:**: `numToSubtractFromEnd` - - UCSC uses interbase numbering, to get to end inclusive numbering you must subtract one from the ends of everything, thus set to 1.

---


### UCSCGeneModelTableReader

```
public UCSCGeneModelTableReader()
```


**Method Detail**

### main

```
public static void main(java.lang.String[] args)
```

---


### printAllExons

```
public void printAllExons(int bpEndMod)
```

:   Prints all exons to standard out. Subtracts bpEndMod from start, adds it to end.

---


### readOutHash

```
public void readOutHash()
```

:   To test your hash.

---


### splitByChromosome

```
public void splitByChromosome()
```

:   Splits a UCSCGeneLine[] by chromosome into a HashMap of chromosome:UCSCGeneLine[].

---


### parseGeneTableFile

```
public void parseGeneTableFile(java.io.File file,
                               int numToSubtractFromEnd)
```

:   **Parameters:**: `numToSubtractFromEnd` - - UCSC uses interbase numbering, to get to end inclusive numbering you must subtract one from the ends of everything, thus set to 1.

---


### getChromSpecificGeneLines

```
public java.util.HashMap<java.lang.String,UCSCGeneLine[]> getChromSpecificGeneLines()
```

---


### getGeneLines

```
public UCSCGeneLine[] getGeneLines()
```

---


### setGeneLines

```
public void setGeneLines(UCSCGeneLine[] geneLines)
```


---


|  |  |  |
| --- | --- | --- |
| **Overview** \| **Package** \| **Class** \| **Use** \| **Tree** \| **Deprecated** \| **Index** \| **Help** | |  |
| **PREV CLASS**   NEXT CLASS | **FRAMES**    **NO FRAMES**     **All Classes** |  |
| SUMMARY: NESTED \| FIELD \| CONSTR \| METHOD | DETAIL: FIELD \| CONSTR \| METHOD |  |


---
